# Surgical technique for development of a clinically-representative ventral hernia repair infection rat model

**DOI:** 10.1016/j.mex.2020.100887

**Published:** 2020-04-22

**Authors:** Albert Thomas Anastasio, Jeffrey L. Van Eps, Joseph S. Fernandez-Moure

**Affiliations:** a Department of Orthopaedic Surgery, Duke University School of Medicine, Durham, NC 27710, United States; b Department of Surgery, Division of Trauma, Acute, and Critical Care Surgery, Duke University School of Medicine, Durham, NC 27710, United States

**Keywords:** Infection, Hernia, Mesh

## Abstract

The animal model of infection following ventral hernia repair (VHR) has previously been utilized in exploring treatments and innovative therapies, such as implantation of biologic mesh imbedded with various anti-bacterial properties. The rat model has been utilized most commonly, but prior work has failed to recreate an adequately clinically representative model of infection following VHR. Additionally, there is lack of standardization of mesh infection severity across existing literature. Therefore, the aim of this paper is to describe the creation of a clinically representative VHR infection model utilizing an index procedure where a hernia defect is created followed by a VHR using biologic mesh and subsequent infectious agent inoculation. Additionally, we describe the development of a standardization index to quantify severity of mesh infection: the Mesh Infection Severity Index (MISI).

• Our protocol involves two procedures, an index procedure where a hernia model is created, and a subsequent procedure where an infectious inoculant is introduced.
• We describe the MISI, a standardization tool we hope will allow for ease of cross-institutional data assessment.
• In summary, our protocol not only serves as a more clinically representative animal model, but also includes a novel metric to standardize mesh infection severity.

## Graphical abstract

**Figure:**
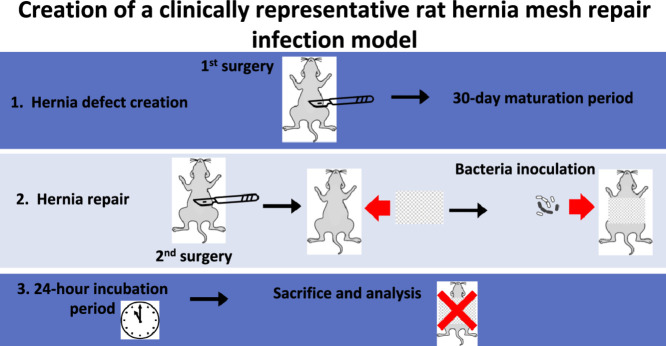

Specifications table

**Table:** 

Subject Area	Medicine and Dentistry
More specific subject area	*Hernia Surgery*
Method name	*Characterization of Hernia Mesh Contamination in a Rat Model of Chronic Hernia Repair*
Name and reference of original method	Majumder, Arnab, Clayton C. Petro, Lijia Liu, Mojtaba Fayezizadeh, and Yuri W. Novitsky. “Development of a novel murine model for treatment of infected mesh scenarios.” *Surgical endoscopy* 31, no. 2 (2017): 922–927.
Resource availability	

## Method details

Ventral hernia repair (VHR) is an extremely common procedure in general surgery, and the incidence continues to increase natonally [1]. Unfortunately, infection following VHR complicates 1%–2% of all procedures utilizing mesh implantation to repair abdominal wall defects [2]. The current standard of care for this complication is mesh explantation, coupled with a course of antibiotics to both achieve source control and eradiate infection [3], [4]. Conservative therapy with nonoperative management and long term antibiotics is typically rendered ineffective given biofilm generation by organisms that typically cause prosthetic mesh infection [9]. The difficulty in treating infections after VHR with mesh and the morbidity associated with the explantation procedure has necessitated a significant emphasis on investigation in reducing bacterial contamination, invasion, and infection of prosthetic mesh [5]. Therefore, a number of different methodologies in various animal models have been developed to explore potential therapeutic avenues.

In 2017, Vogels et al. provided a critical review of all available animal models for abdominal wall hernia research [6]. Current animal models include pig (16.8%), rat (53.3%), mice (3.5%), rabbit (21.0%), guinea pig (2.2%), and other animal models (3.2%) [6]. With rats comprising the majority of VHR animal models, a number of different methodologies have been described for the creation and evaluation of the VHR rat model. The three most frequently used methodologies are those published by Alponat et al. (12 studies) [7], Peter-Puchner et al. (4 studies) [8], and Klinge et al. (3 studies) [9]. The Alponat et al. methodology was by far the most commonly followed rat model for VHR, appearing in 12 of the analyzed studies. This methodology includes a single operative procedure, where a hernia is created, and a mesh repair is undertaken – all within the same anesthesia event [7]. While this methodology has been validated, it fails to adequately recreate the ventral hernia clinical scenario, where hernia sac tissue has had time to form adhesions and mature. A single operative procedure where a defect is both made and repaired fails to capture the complexity of this situation.

In the Vogels et al. report of animal models for hernia repair, 9.5% of the studies analyzed involved creation of an infection model. Prior methodologies for the creation of a VHR infection rat model predominately utilized a subcutaneously implanted biologic mesh which is then inoculated with an infectious agent. [10] Such a methodology is described by Bellows et al. [10], where various concentrations of methicillin-resistant *Staphylococcus aureus* were injected into previously subcutaneously implanted biologic mesh acellular human dermis (ADM) and porcine small intestine submucosa (SIS).[10] A subcutaneously implanted biologic mesh that has subsequently been inoculated with an infectious agent inadequately recreates the clinical scenario, given lack of initial hernia pathology.

Thus, our authors aimed to create a superior model of infection following VHR. To do so, we chose to perform two procedures – an index procedure where a ventral hernia defect was created, and a treatment procedure where a biologic mesh was implanted to repair the defect after a period of time had elapsed to allow the defect to materialize. The mesh was then inoculated with infectious agent. To evaluate the effect of a NO-releasing silica nanoparticle (NOSi) bearing mesh on enhanced Methicillin-resistant *Staphylococcus aureus* (MRSA) killing in an *in vivo* model of mesh infection, we then created a scoring system which we termed the Mesh Infection Severity Index (MISI). This index evaluated mesh adhesion severity, adhesion surface area, abscess formation, skin erythema, and mesh contracture and assigned a composite score based on these subcategories. We believe this methodology to be the most robust and clinically representative VHR infection rat model to date.

## Animals

Sixty-three male Lewis rats (300–315 g) are housed at Houston Methodist Hospital Research Institute (HMHRI) animal facility, with strict adherence to the National Research Council's *Guide for the Care and Use of Laboratory Animals* (Protocol # AUP-1012-0047). Clearance and monitoring of the study are obtained from the HMHRI Institutional Animal Care and Use Committee. Lewis rats are chosen because of their inbred nature (Charles River; Wilmington, Mass.) as ideal candidates for our VHR infection model. Animals are examined daily and food and water are replenished as needed. All health issues are addressed appropriately throughout the duration of the study. Rats are divided into three study groups using a random number generator. One group undergoes ventral hernia repair (methodology described below) with Parietex™-PCO (Covidien Medtronic, Minneapolis, MN). A second group receives a PCO mesh and bare silica particles and a third group (test group) receives a *N*-diazeniumdiolate silica (NOSi) treated PCO mesh. All rats receive an index procedure where a ventral hernia defect was created. The initial procedure is followed by subsequent mesh repair and inoculation with concentrations of either 1 × 10^4^, 1 × 10^6^, or 1 × 10^8^ MRSA inoculant.

## Abdominal wall defect creation

**Fig. 1:**
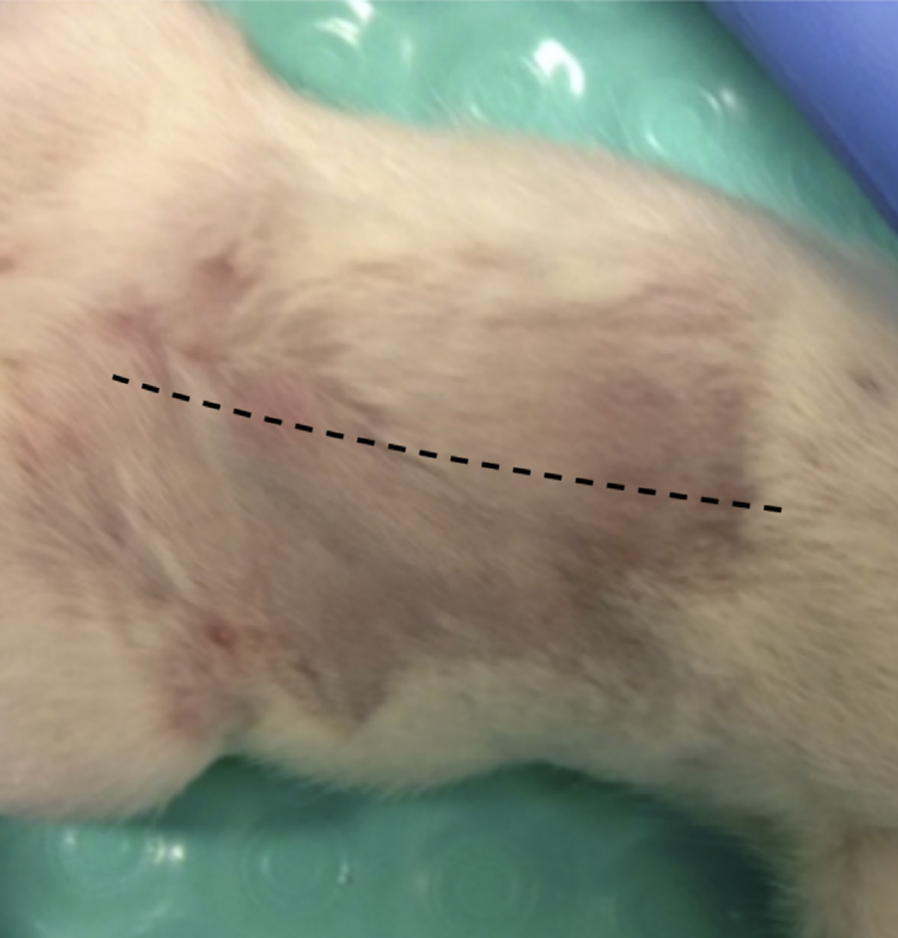
After analgesia administration (Buprenorphine, 0.03 mg/kg and Carprofen, 5 mg/kg) and anesthesia induction (2.5%–3.0% Isoflurane/oxygen mixture), all rats undergo a 3 cm midline skin incision across the abdomen. There is strict adherence to sterile technique throughout the procedure.

After administration of Buprenorphine (0.03 mg/kg) and Carprofen (5 mg/kg) for preoperative analgesia, anesthesia is induced and maintained using a 2.5%–3.0% Isoflurane/oxygen mixture through a non-rebreather mask. All rats are then shaved and sterilized using chlorhexidine gluconate solution. A sterile field is then created using sterile surgical drapes. Proper aseptic technique is used for the duration of all procedures. All rats then undergo a 3 cm midline skin incision (Fig. 1).

**Fig. 2:**
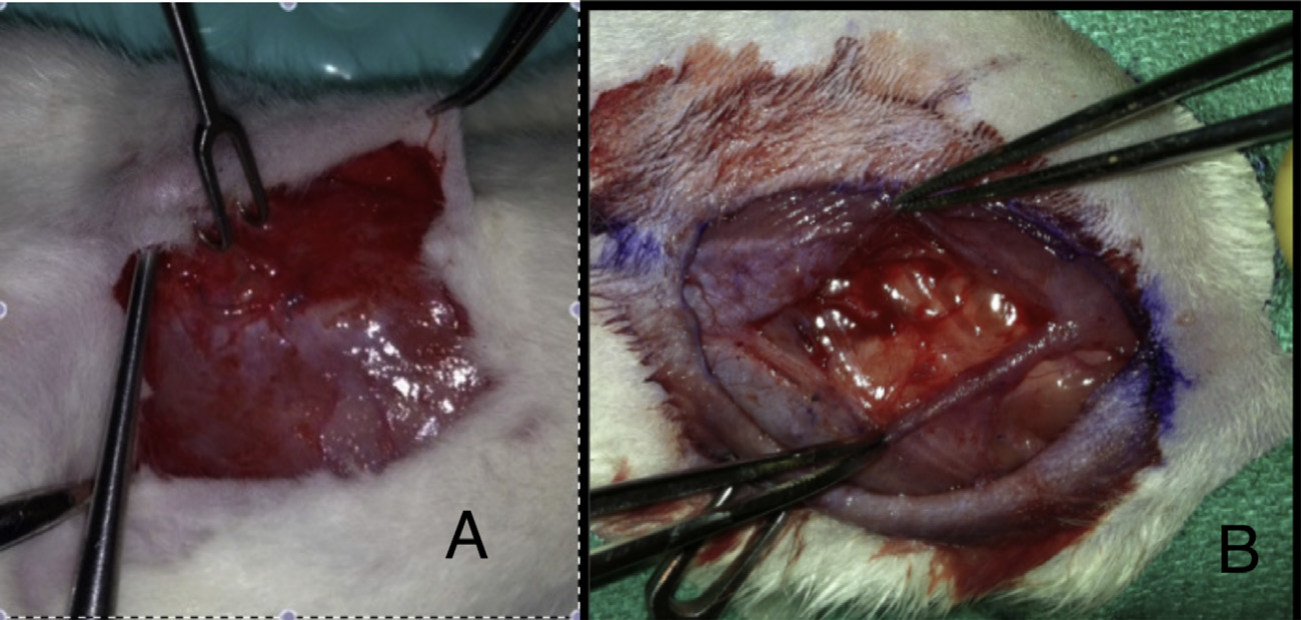
The index procedure involves creation of a fascial defect followed by skin approximation and stapling. A: Sharp dissection is then performed to expose the fascial layer. B: A 2 cm incision in the fascial layer is created, and this is followed by closure of the skin without re-approximation of the fascial defect.

After the initial incision, skin flaps are raised using sharp dissection. A full thickness abdominal midline fascia/muscle/peritoneum defect measuring 2 cm in length is then made (Fig. 2). The defect is left unrepaired and the skin is then approximated and stapled. Buprenorphine (0.03 mg/kg) and Carprofen (5 mg/kg) are then administered for postoperative analgesia. To preserve incision integrity, Elizabethan collars are applied for the first 5 postoperative days and staples are removed after 14 days.

**Fig. 3:**
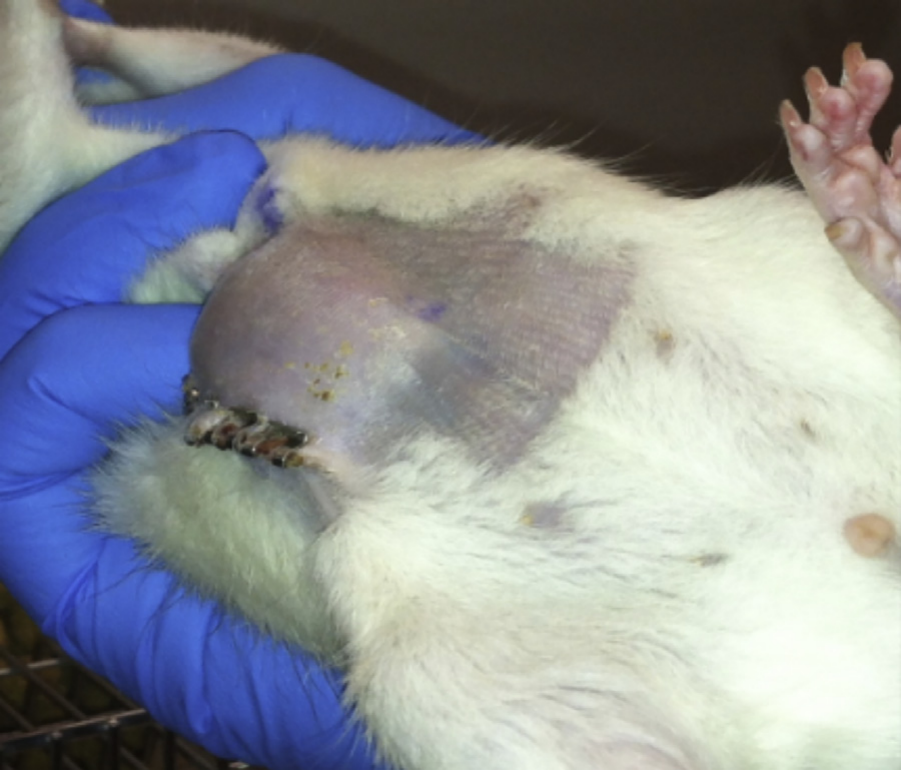
After the initial procedure and creation of an abdominal wall defect, rats exhibit significant “hernia bulge” indicating a successful recreation of a ventral hernia.

After the index procedure and creation of an abdominal wall fascial defect, 28 days are allocated for maturation of the defect. Physical exam is performed, and weights are obtained daily for 10 days, and then weekly thereafter. After 28 days, rats are again examined to ensure successful creation of a ventral hernia model (Fig. 3).

## Abdominal wall defect repair and inoculation

After an index procedure where an abdominal wall defect is created and following a 28-day maturation period, the treatment procedure is then undertaken. Rats undergo hernia repair with either PCO mesh alone, PCO mesh and bare silica particles (Si-PCO), or NOSi treated PCO mesh (NOSi-PCO). With regards to preparation of the Si-PCO and NOSi-PCO meshes, each had 66 mg of Si and NOSi respectively loaded onto the mesh material. This quantity was chosen because this was the mass of NOSi particles that resulted in no bacterial colony survival at the concentration 1 × 10^4^.

**Fig. 4:**
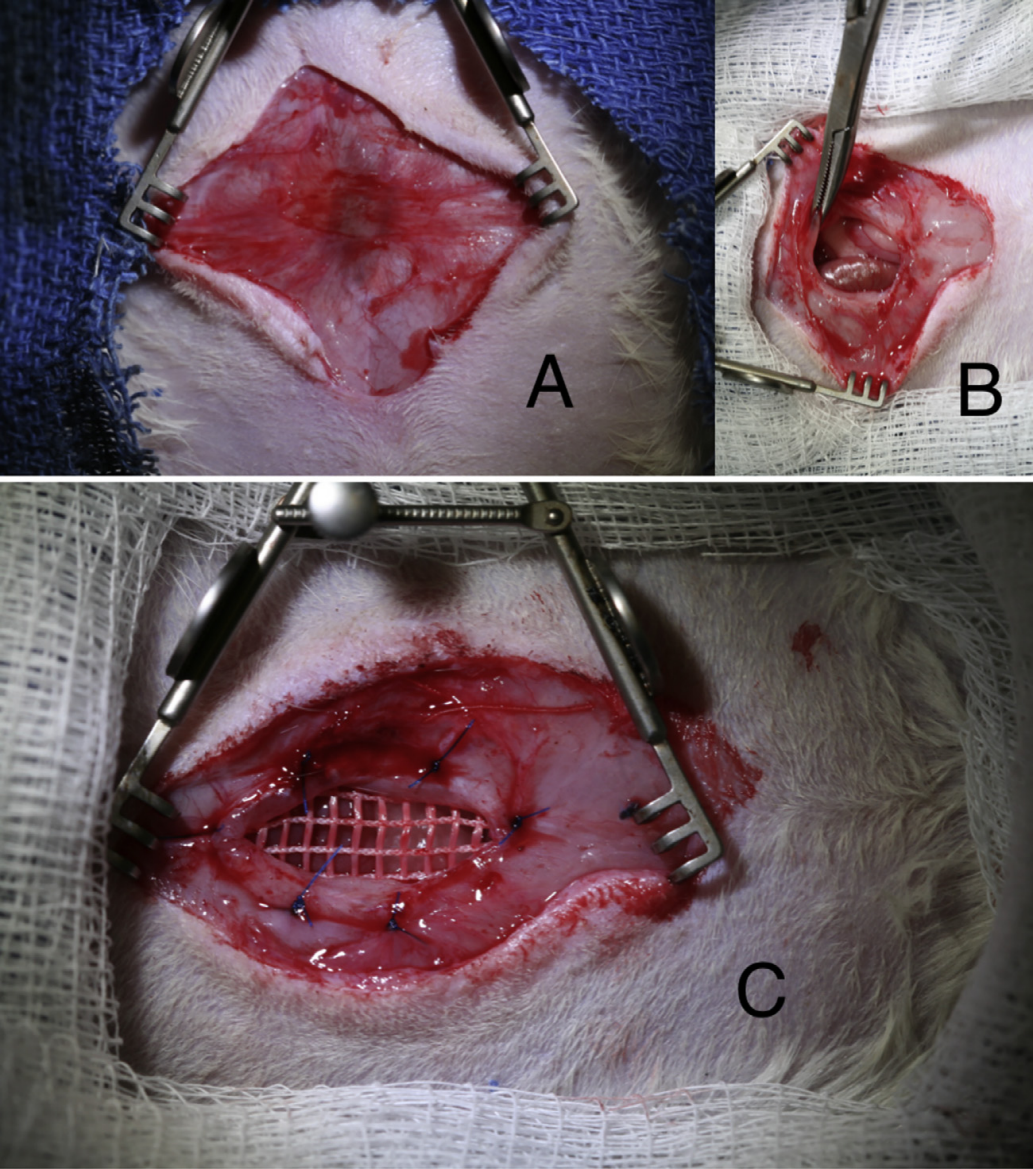
Ventral hernia repair surgical technique. Preoperative analgesia and anesthesia induction are achieved. A: Skin incision is made to expose the hernia sac. B: Through sharp dissection, the protruding viscera are exposed, and fascial edges are defined. C: A 2.5 × 1.5 cm piece of mesh is used to repair the previously created 2 cm abdominal wall defect, allowing for 5 mm of overlap between muscle and mesh.

Preoperative analgesia is once again administered using Buprenorphine (0.03 mg/kg) and Carprofen (5 mg/kg). Anesthesia induction is carried out using a 2.5%–3.0% Isoflurane/oxygen mixture through a non-rebreather mask. Sterile prepping and draping are carried out to ensure aseptic conditions prior to inoculation of MRSA. A skin incision is made to expose the hernia sac (Fig. 4A). Sharp dissection is utilized to expose and free the protruding viscera and the fascial edges are then defined (Fig. 4B). In order to allow for 5 mm of overlap between muscle and mesh, a 2.5 × 1.5 cm piece of mesh is used to repair the previously created 2 cm abdominal wall defect. 5-0 Prolene ® (Ethicon, Somerville, NJ) sutures are spaced equally apart to achieve fascial fixation and an intraperitoneal bridging repair is undertaken (Fig. 4C).

**Fig. 5:**
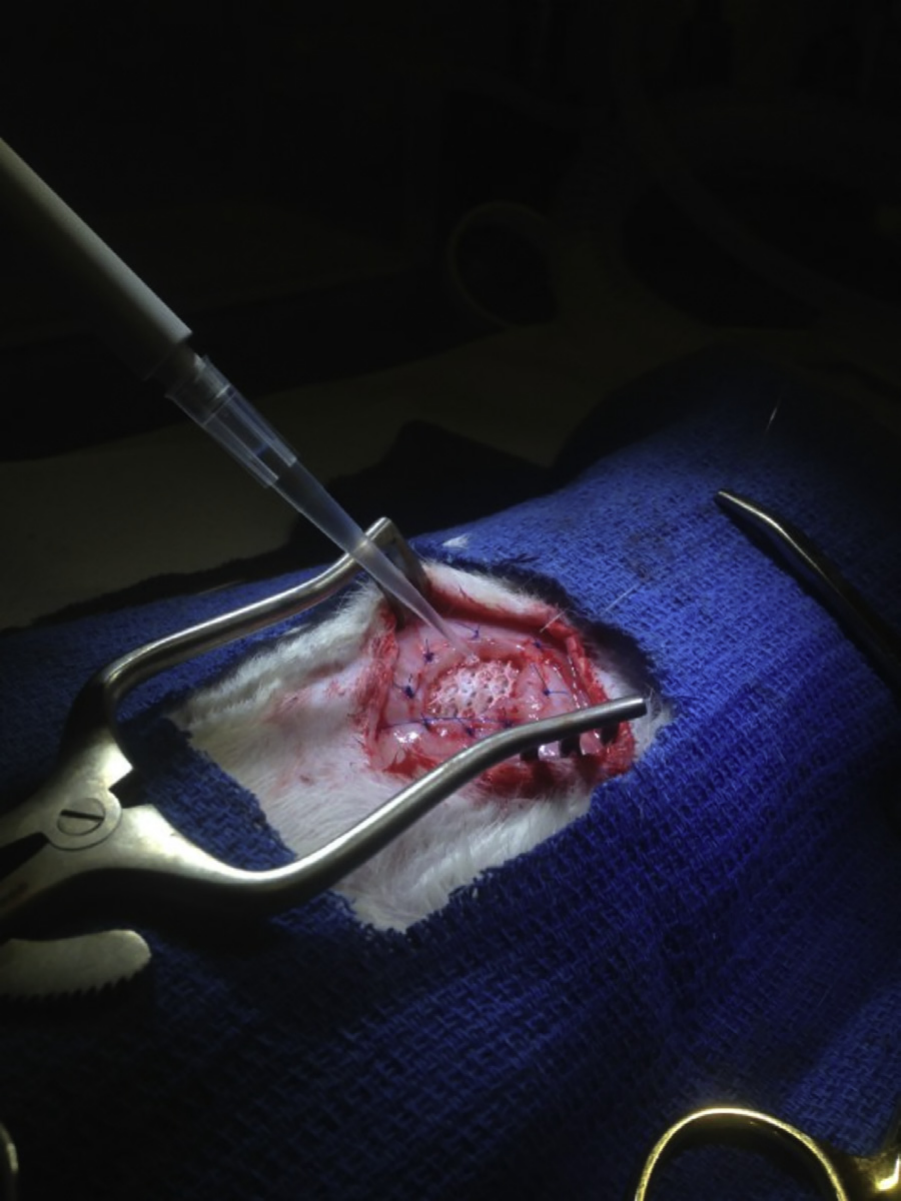
Inoculation of infectious agent. 1 × 10^4^, 1 × 10^6^, or 1 × 10^8^ MRSA is applied directly to the exposed anterior surface of the mesh in a volume of 50 μl (Fig. 5) prior to skin closure during the VHR procedure.

After bridging mesh repair of the previously created abdominal wall defect, 1 × 10^4^, 1 × 10^6^, or 1 × 10^8^ MRSA is then applied directly to the exposed anterior surface of the mesh in a volume of 50 μl (Fig. 5). Skin is then closed using a 4-0 Monocryl ® (Ethicon, Somerville, NJ) running subcuticular stich. 3M™ Vetbond™ Tissue Adhesive (3 M Animal Care Products, St. Paul, MN) is then utilized to further seal the incision, thus ensuring maintenance of bacterial solution within the surgical area. Elizabethan collars are then reapplied to the rats to secure the repair from animal manipulation. Buprenorphine (0.03 mg/kg) and Carprofen (5 mg/kg) are administered for postoperative analgesia.

**Fig. 6:**
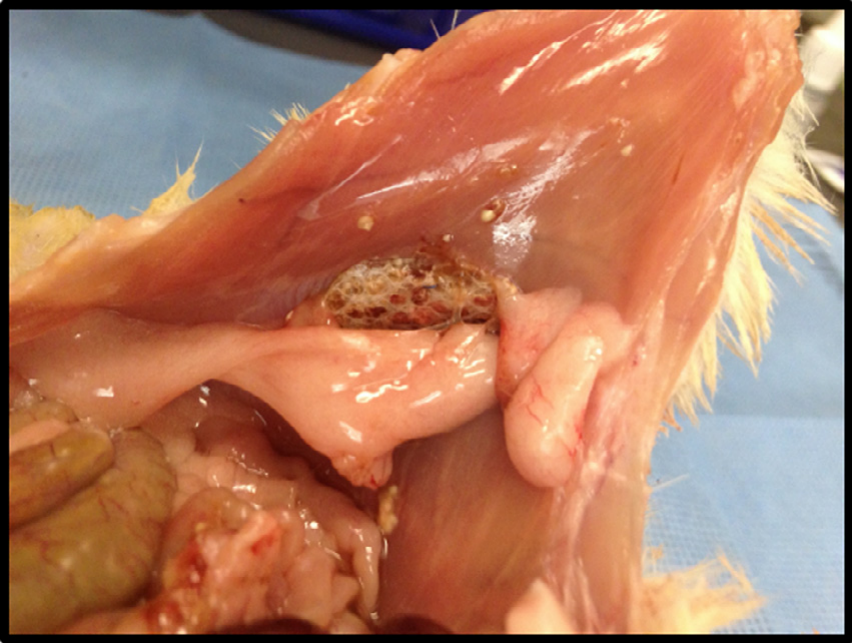
Visualization of ventral hernia repair mesh 24 h after inoculation of MRSA containing-solution. Mesh adhesion severity, adhesion surface area, abscess formation, and mesh contracture are scored based on post-dissection findings. These scores are combined with a skin erythema score to generate a final composite score termed the Mesh Infection Severity Index (MISI).

## Euthanization and specimen harvest

After the treatment procedure and subsequent MRSA inoculation, a 24-h incubation period is exploited to allow for bacterial spread. Using 70% inhaled carbon dioxide, animals are then euthanized, and asystole is confirmed via thoracotomy. The presence or absence of skin erythema is recorded. Following confirmation of death, the abdomen is then entered with a C shaped incision around the mesh and is subsequently elevated to allow for inspection and grading (Fig. 6). A composite score termed the Mesh Infection Severity Index (MISI) was developed and is comprised of the following parameters, which each received their own equally weighted score: mesh adhesion severity, adhesion surface area, abscess formation, skin erythema, and mesh contracture (Table 1).

**Table 1:** Mesh Infection Severity Index.

Adhesion Severity	0 = no adhesion
	1 = thin film and no visible vesselsg
	2 = moderate thickness film and visible vessel
	3 = thick adhesion and visible vessel

Adhesion Surface Area	0 = <25%
	1 = 25-50%
	2 = 50-75%
	3 = >75%

Abscess	0 = none
	1 = present

Skin Erythema	0 = none
	1 = present

Mesh Contracture	0 = none
	1 = present

Scoring of the erythema and VHR quality parameters is carried out by at least two blinded observers. Two previously validated indices from the Surgical Membrane Study Group and Linsky et al. documenting the severity of adhesion as well as the percentage of mesh covered by intraperitoneal contents (i.e., intestine, solid organ, or omentum) [11], [12] are utilized in adhesion scoring. After scoring of the adhesions, scar tissue is lysed and the mesh is assessed for contracture or abscess formation. The presence of abscess formation is defined by the visualization of necrotic tissue or debris, purulence or obvious fluid collection in association with the mesh. Specimens are flash frozen and contained in the freezer for further analysis.

## Method validation

This surgical technique of ventral hernia creation followed by mesh repair and subsequent inoculation with MRSA resulted in an effective model to investigate various adjuncts to VHR utilizing a biologic mesh in reducing susceptibility to infection post-operatively. This methodology is validated in our recent submission comparing Parietex™-PCO mesh, PCO mesh and bare silica particles, or *N*-diazeniumdiolate silica treated PCO mesh in a VHR infection rat model.

## Declaration of Competing Interest

Nothing to declare.
